# Within-colony feeding selectivity by a corallivorous reef fish: foraging to maximize reward?

**DOI:** 10.1002/ece3.778

**Published:** 2013-09-20

**Authors:** Rohan M Brooker, Geoffrey P Jones, Philip L Munday

**Affiliations:** 1 School of Marine and Tropical Biology, James Cook UniversityTownsville, Queensland, Australia; 2 ARC Centre of Excellence for Coral Reef studies, James Cook UniversityTownsville, Queensland, Australia

**Keywords:** *Acropora*, corallivory, optimal foraging theory, *Oxymonacanthus longirostris*, predator–prey interactions, prey morphology

## Abstract

Foraging theory predicts that individuals should choose a prey that maximizes energy rewards relative to the energy expended to access, capture, and consume the prey. However, the relative roles of differences in the nutritive value of foods and costs associated with differences in prey accessibility are not always clear. Coral-feeding fishes are known to be highly selective feeders on particular coral genera or species and even different parts of individual coral colonies. The absence of strong correlations between the nutritional value of corals and prey preferences suggests other factors such as polyp accessibility may be important. Here, we investigated within-colony feeding selectivity by the corallivorous filefish, *Oxymonacanthus longirostris*, and if prey accessibility determines foraging patterns. After confirming that this fish primarily feeds on coral polyps, we examined whether fish show a preference for different parts of a common branching coral, *Acropora nobilis*, both in the field and in the laboratory experiments with simulated corals. We then experimentally tested whether nonuniform patterns of feeding on preferred coral species reflect structural differences between polyps. We found that *O. longirostris* exhibits nonuniform patterns of foraging in the field, selectively feeding midway along branches. On simulated corals, fish replicated this pattern when food accessibility was equal along the branch. However, when food access varied, fish consistently modified their foraging behavior, preferring to feed where food was most accessible. When foraging patterns were compared with coral morphology, fish preferred larger polyps and less skeletal protection. Our results highlight that patterns of interspecific and intraspecific selectivity can reflect coral morphology, with fish preferring corals or parts of coral colonies with structural characteristics that increase prey accessibility.

## Introduction

Animals seldom exist within a nutritionally homogeneous environment, and as a result of variable nutritional composition and prey accessibility, they can experience a range of dietary options (Rapport 1980). Optimal foraging theory predicts that an individual should prefer prey species of high nutritional value relative to the energy spent to locate, capture, and consume the prey (Charnov 1976; Pyke et al. 1977). Differences in the nutrient composition of prey can play a key role in determining species-specific preferences (Jensen et al. 2012). However, the nutritional value of a given prey species may vary in response to differences in the condition or reproductive status of individuals, making optimum prey choice difficult (Fitzgibbon 1990; Gende et al. 2001; Lane et al. 2011). The relative accessibility, or vulnerability, of different prey species may also be important (Harder 1983; Hoogland et al. 2006; Plath et al. 2011). The presence of antipredator defenses or morphological features that constrain feeding can increase the time required to locate, manipulate, and consume food (Werner and Hall 1974; Temeles et al. 2009), reducing their value. Relative nutritional value may also vary within an individual, with consumers selectively targeting specific parts that provide the greatest nutritional benefit (Andrew and Jones 1990; Gende et al. 2001; Pekar et al. 2010; Pitman and Durban 2012) or the least protected parts of a prey organism.

On coral reefs, many of the associated fishes are dependent on live corals, as food, for shelter, or during recruitment (Munday et al. 2008). Coral-feeding fishes are among the most specialized species found on coral reefs, selectively consuming corals from particular genera or species (Berumen et al. 2005; Pratchett 2007; Cole et al. 2008, 2010; Rotjan and Lewis 2008; Brooker et al. 2013). The underlying basis of this selectivity is not well understood but could relate to a variety of factors such as biochemical composition, morphology, or antipredator defenses. It has often been assumed that selectivity relates to variation in the nutritional value between corals (Pisapia et al. 2012), and recent studies have shown that consuming a preferred coral can have positive effects on corallivorous fishes, improving relative growth rates (Berumen and Pratchett 2008), body condition (Berumen et al. 2005; Brooker et al. 2013), and reproductive output (Brooker et al. 2013). However, the few studies that have attempted to relate the biochemical profiles of coral tissue, in particular the levels of energetic macronutrients, to corallivore preferences have failed to find strong correlations with fitness-related benefits (Tricas 1989; Keesing 1990; Rotjan and Lewis 2005; Pisapia et al. 2012). Furthermore, patterns of coral-feeding within different coral species have received little attention, and it is not known if coral-feeding fishes target specific parts of the coral colony, either due to nutritional variation of differences in prey accessibility.

Scleractinian corals are generally composed of colonies of individual polyps, all extending from an aragonite exoskeleton. The basic anatomy of a coral polyp is relatively simple, consisting of a gastrointestinal chamber enclosed by a tentacle-ringed mouth. Each polyp produces an individual exoskeletal cup, the corallite, that provides protection for the polyp (Klaus et al. 2007). Polyps are connected by gastrovascular canals that run through the thin layer of interpolyp tissue, the coenosarc. Exoskeletal structure and polyp morphology vary extensively both between- and within-coral taxa (Klaus et al. 2007; Todd 2008), and this variation could affect how efficiently coral tissue can be consumed. For example, by selectively foraging on the coral *Pocillopora meandrina*, a species with clustered polyps, the butterflyfish, *Chaetodon multicinctus* increased its calorific intake per bite relative to when foraging on other corals (Tricas 1989). If corallivores attempt to maximize their efficiency when foraging, then preferences for specific corals may therefore reflect their morphological traits. To date, studies of corallivory and corallivore foraging preferences have generally considered each coral species to be an independent prey type (Cole et al. 2008) and have not tested whether corallivores use these corals uniformly or are influenced by factors, such as biochemical or morphological variation, that may occur within a single coral (but see Rotjan and Lewis 2009). Investigating prey selection at this finer scale may help define the processes driving prey selection in corallivorous fishes.

**Figure 1:**
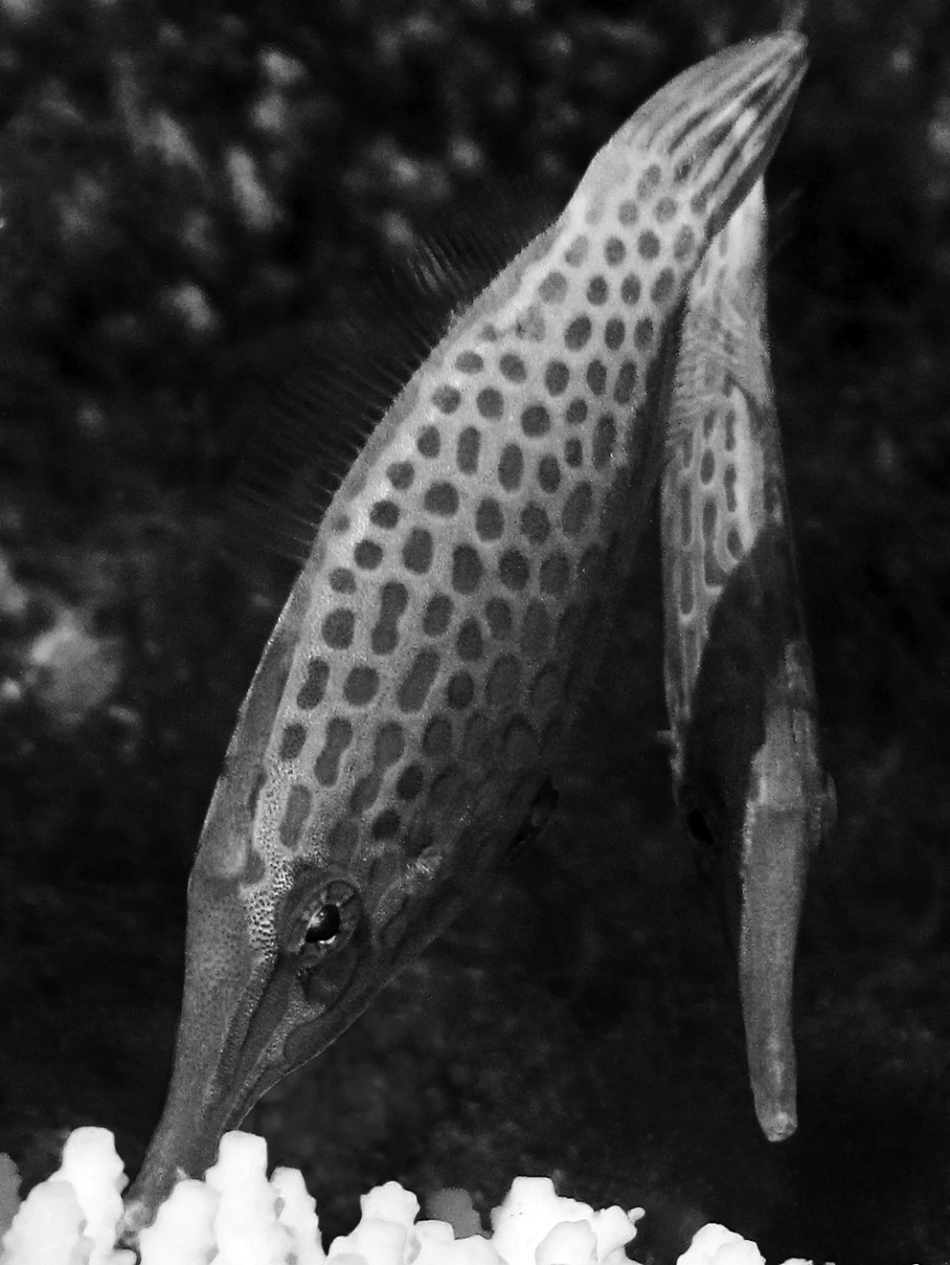
The harlequin filefish, *Oxymonacanthus longirostris*, feeding on *Acropora* coral. Photo: R. M. Brooker.

The objective of this study was to investigate, for the first time, the relative roles of nutrition and polyp accessibility in determining within-colony feeding selectivity by the corallivorous filefish, *Oxymonacanthus longirostris* (Bloch & Schneider, 1801; Fig. 1). This filefish is an obligate corallivore that feeds almost exclusively on corals from the genus *Acropora* (Kokita and Nakazono 2001). On the southern Great Barrier Reef (GBR), it primarily feeds on *Acropora nobilis* (Dana, 1846), which is an abundant branching coral in that region (Veron 2000). However, it also exhibits a strong dietary preference for *Acropora millepora* (Ehrenberg, 1834) and other less abundant coral species (Brooker et al. 2013). Patterns of feeding within these coral species are unknown. Here, we specifically set out to (1) confirm that *O. longirostris* primarily feeds on coral polyps; (2) determine whether or not *O. longirostris* shows a preference for different parts of *A. nobilis* coral colonies in the field and whether this is related to polyp density or corallite structure; (3) compare feeding patterns to determine whether food accessibility determines foraging location and whether fish are able to modify feeding patterns in response to food accessibility; and finally, (4) experimentally test whether nonuniform patterns of feeding on preferred coral species (*A. millepora* and *Acropora tenuis* [Dana, 1846]) reflect structural differences between polyps that may affect foraging efficiency.

## Methods

### Study species and sites

The harlequin filefish, *Oxymonacanthus longirostris* (Monacanthidae), is distributed on shallow coral reefs throughout the Indo-Pacific and can be found in low numbers in sheltered areas of the GBR with high coral cover. A preliminary aquarium observational study was conducted at Lizard Island, northern GBR (14°40′S; 145°27′E), to establish that *O. longirostris* feeds on coral polyps. The field components of this study were conducted at Big Peninsula Reef, Great Keppel Island (GKI) on the southern GBR, Australia, during September 2009. GKI (23°10.7′S; 150°57.6′E) is a large continental island surrounded by reefs dominated by branching *Acropora nobilis*. Two aquarium choice experiments were conducted: one at Reef HQ Aquarium, Townsville, Australia in July 2010 and the other at the JCU Research Aquarium, Townsville, Australia in October 2011.

#### What coral structures are targeted by *Oxymonacanthus longirostris*?

Although it is generally assumed that *O. longirostris* is a coral polyp predator, this has not been quantified. To determine whether *O. longirostris* do target coral polyps, or alternatively feed on interpolyp tissue (coenosarc), or feed indiscriminately across the coral surface, an observational study of foraging activity was conducted. Trials took place within a circular tank (1.5 m diameter) constantly supplied with fresh sea water and aeration to maintain water quality. Twelve *O. longirostris* were kept in the tank. Coral skeleton was placed along the perimeter to provide structural complexity and reduce stress to the fish but the center of the tank was kept clear. An underwater video camera (GoPro, Woodman labs ltd.) was placed at one end of the tank. For each foraging trial, a fragment of *A. millepora* composed of approximately 3 branches was placed 20 cm in front of the camera, held upright at a natural angle. All foraging was recorded for a period of 5 min, with fish generally beginning to forage within several seconds of the fragment being introduced. Footage from each trial was then analyzed frame by frame with the location of the first 30 bites recorded. Only bites where both a fish's mouth and the coral surface were clearly visible were included. Bite locations were recorded as either directly on a polyp or on the coenosarc. Differences between bite locations were determined using an independent t-test.

#### Field study of selectivity within *Acropora nobilis* colonies

A field study was conducted to determine whether *O. longirostris* feeds on prey coral colonies uniformly. As this species is diurnally active, foraging observations were conducted between 09:00 and 16:00. Twenty haphazardly chosen individuals were followed for 10 min periods with the location of all bites on the coral *A. nobilis* recorded. This coral has an open branching morphology allowing for accurate recording of bite locations and is highly abundant at the study site where it forms the bulk of *O. longirostris* diet (Brooker et al. 2013). Each branch that an individual was observed foraging on was divided into three equal sections by the observer, recorded as top, central, and base. Observations were conducted on SCUBA with fish followed at 2–3 m distance. At this distance, fish exhibited no signs of disturbance. Observations began when fish commenced foraging, taken as indication of acclimation of the fish to the diver's presence. Data were analyzed using univariate analyses of variance (ANOVA) with post hoc pairwise comparisons conducted using the Tukey HSD test.

#### Experimental test of preferences using simulated *Acropora nobilis* branches

A series of aquarium experiments were conducted using simulated *A. nobilis* branches to further determine the role that food accessibility plays in foraging decisions. Each simulated coral branch consisted of three separate 4 × 1.5 cm cylindrical segments, constructed of an inert polymer, threaded onto a central stand. Thus, when assembled, each simulated coral branch formed a 12 × 1.5 cm cylinder extending vertically into the water column with a base, middle, and tip. A series of 28 artificial corallites, constructed of 1.5 mm diameter plastic tubing, were fixed on to each cylindrical segment. Three distinct artificial corallite lengths were constructed; shallow 1.0 mm, medium 1.5 mm, and deep 2.0 mm, corresponding to three levels of food accessibility analogous to thecal wall extension. Each section had only one length of artificial corallite. In this arrangement, all combinations of position and accessibility could be tested. Food used in these experiments consisted of a homogenous mix of finely puréed prawn meat bound with gelatin. The fine consistency of this mix allowed the biochemical composition and amounts of food used in each trial to be standardized while gelatin component prevented dissipation during trials. Fish used in this experiment were acclimated to this food in addition to live coral tissue and were actively accepting it by the start of experimentation. Prior to each trial, 0.1 mL of food was injected into the base of each corallite using a hypodermic needle. In this way, while fish were able to access food at all depths, food was closer to the surface of shorter artificial corallites and therefore assumed to require less effort to search for and acquire. During the experimental period, fish were kept in individual 100 L circular plastic tanks supplied with constant fresh water and oxygenation. Tanks had black, nonreflective interiors to reduce stress to the fish. For each trial, the appropriately arranged simulated coral was placed at the center of the tank. All bites, along with their location, were then recorded for a 10-min period following the first bite. Fish were not able to see observer during the trials. Experiment one examined the relationship between foraging selectivity and branch location. During this experiment, all three segments of the simulated coral branch had the same artificial corallite depth. All fish were run through each of the three corallite depths in a randomized order with one trial per day. Experiment two examined the relationship between foraging selectivity and food accessibility. During this experiment, each simulated coral branch had one segment of each artificial corallite depth. Each fish was run through each potential combination of these three depths in a randomized order with one trial per day. Due to nonindependence between segments, data for each simulated coral branch experiment were analyzed using a nonparametric Kruskal–Wallis test. Post hoc pairwise comparisons were conducted using Dunn's procedure (Dunn 1964) with a Bonferroni correction for multiple comparisons.

#### Aquarium study of within-colony selectivity

An aquarium choice experiment was conducted to further examine how foraging varies within corals and whether selectivity relates to position of structural characteristics. This experiment consisted of a series of pairwise trials where fish were offered a choice between two coral fragments from two different *Acropora* species and two points of origin (top or bottom sections of branches). The two *Acropora* species used, *A. millepora* and *A. tenuis,* have similar digitate colony morphologies along with similar individual branch sizes; however, they appear to vary with regard to corallite morphology depending on location along a branch. An initial pairwise choice experiment was conducted to determine whether fish exhibited a general foraging preference between these species. For this experiment, relatively large (15 cm diameter) fragments were chosen as they included a number of individual branches removing any branch effect. Once the presence or absence of a foraging preference was established, fish were run through six randomly ordered trials using smaller fragments representing all combinations of both *Acropora* species and points of origin (top or bottom sections of branches). For each fish, one trial was conducted per day over 6 days.

*Oxymonacanthus longirostris* were collected from midshelf reefs off Cairns, Australia and held at Reef HQ aquarium, Townsville, Australia. Prior to the commencement of experiments fish were fed *ad libitum* with pieces of the *Acropora* species used in trials supplied in equal abundance supplemented with a standard conditioning diet consisting of prawn meal. This diet maintained fish condition and foraging responses while preventing any learned foraging behavior for specific coral species. During the experimental period, fish were not fed outside of trials and were each kept in independent enclosures to ensure all fish were run through all treatments. Experiments were conducted in a circular enclosure (80 cm diameter) placed within a larger flow through tank (1.2 × 1.2 × 0.5 m) that was supplied with natural light and lined with coral sand. Coral fragments were removed from colonies collected from Pioneer Bay, Orpheus Island, Australia (18°36′S; 146°29′E) and housed at Reef HQ aquarium. As intraspecific variation between corals may affect preferences, randomly selected fragments were taken from three separate colonies of both species. Fragments were removed using needle nose pliers that caused minimal physical damage to tissue and then kept in constantly flowing seawater for 24 h prior to trials to allow for initial recovery from mechanical stress. Fragments where any tissue necrosis occurred were not used. Fragments were placed within the experimental enclosure at two haphazardly selected, opposing points 15 cm from the enclosures edge. Fragments were held upright within a plastic cap using a synthetic rubber compound, minimizing any handling or direct contact with fragments prior to the start of trials. Once placed, fragments were left to acclimate for 20 min.

Individual fish were introduced into a 20 cm diameter mesh cylinder in the center of the experimental enclosure and allowed to acclimate for 10 min. The enclosure allowed fish to observe each fragment and did not restrict sensory cues. At the end of the acclimation period, at which time fish were not displaying stress coloration and were actively swimming, the cylinder was slowly removed allowing fish access to the coral fragments. Foraging behavior was recorded for 10 min after the first bite was taken. Fish were not able to see the observer during trials. Each fragment was used only once as prior foraging may influence how attractive a fragment is to subsequent fishes. The number of bites on each fragment was converted into a percentage of the total taken during a trial. Due to nonindependence between fragments, intraspecific selectivity pairwise trials were analyzed using the nonparametric Wilcoxon signed-rank test.

#### Evaluation of intraspecific morphological variation in corals

The relative importance of structural variation within and between corals species was assessed by comparing fragments that varied in skeletal morphological variables that affect the underlying surface complexity, namely polyp size and density. These were corallite cup diameter across the widest axis of the theca, thecal extension from highest point of the theca to base of the septa, and intercorallite distance between the focal corallite and its nearest neighbor. Morphological variation was assessed between top, middle, and base sections of *A. nobilis* and top and base sections of *A. millepora* and *A. tenuis* to correspond with observational and experimental data. Morphological variance was determined by taking physical measurements of 10 randomly selected corallites on each section of five coral fragments of each species. Morphological variation for each species was then analyzed using multivariate analysis of variance (MANOVA).

## Results

### What coral structures are targeted by *Oxymonacanthus longirostris*?

Experimental observations showed that *O. longirostris* primarily feed on coral polyps with more bites taken directly on polyps (mean = 90.9 ± 1.3) than on coenosarc (mean = 9.1 ± 1.3), a significant difference of 81.8 (95% CI, 78–85.5), (*t*_28_ = 45.2, *P* ≤ 0.001). It is likely that *O. longirostris* consumes the tissue of corals and not mucus or other by-products as polyps were visibly removed following bites.

### Field study of within *Acropora nobilis* colony selectivity

**Figure 2:**
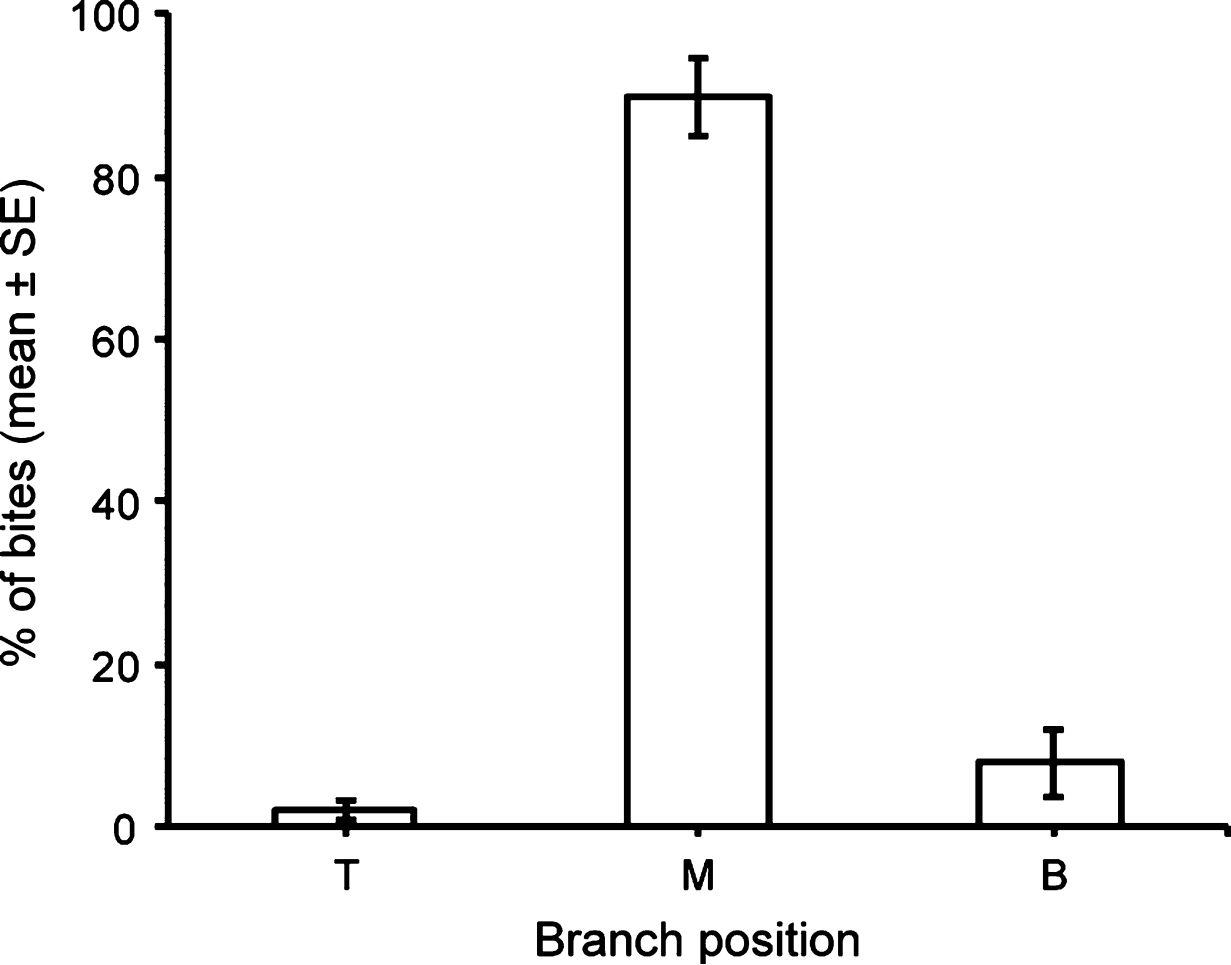
Percentage of total bites (mean ± SE) taken by *Oxymonacanthus longirostris* at different points along branches of *Acropora nobilis* during 10-min feeding observations. Individual branches were divided equally into three sections defined as top (T), middle (M), and base (B). Number of observations = 20.

During field observations, fishes did not forage on *A.nobilis* uniformly (ANOVA, *F*_2,57_ = 164.2, *P* < 0.001). Tukey HSD post hoc comparisons showed that fish took a significantly higher percentage of bites on the central section of branches than either the top and base sections. There were no differences in the percentage of bites taken from top or base sections (Fig. 2).

### Experimental test of preferences using simulated *Acropora nobilis* branches

**Figure 3:**
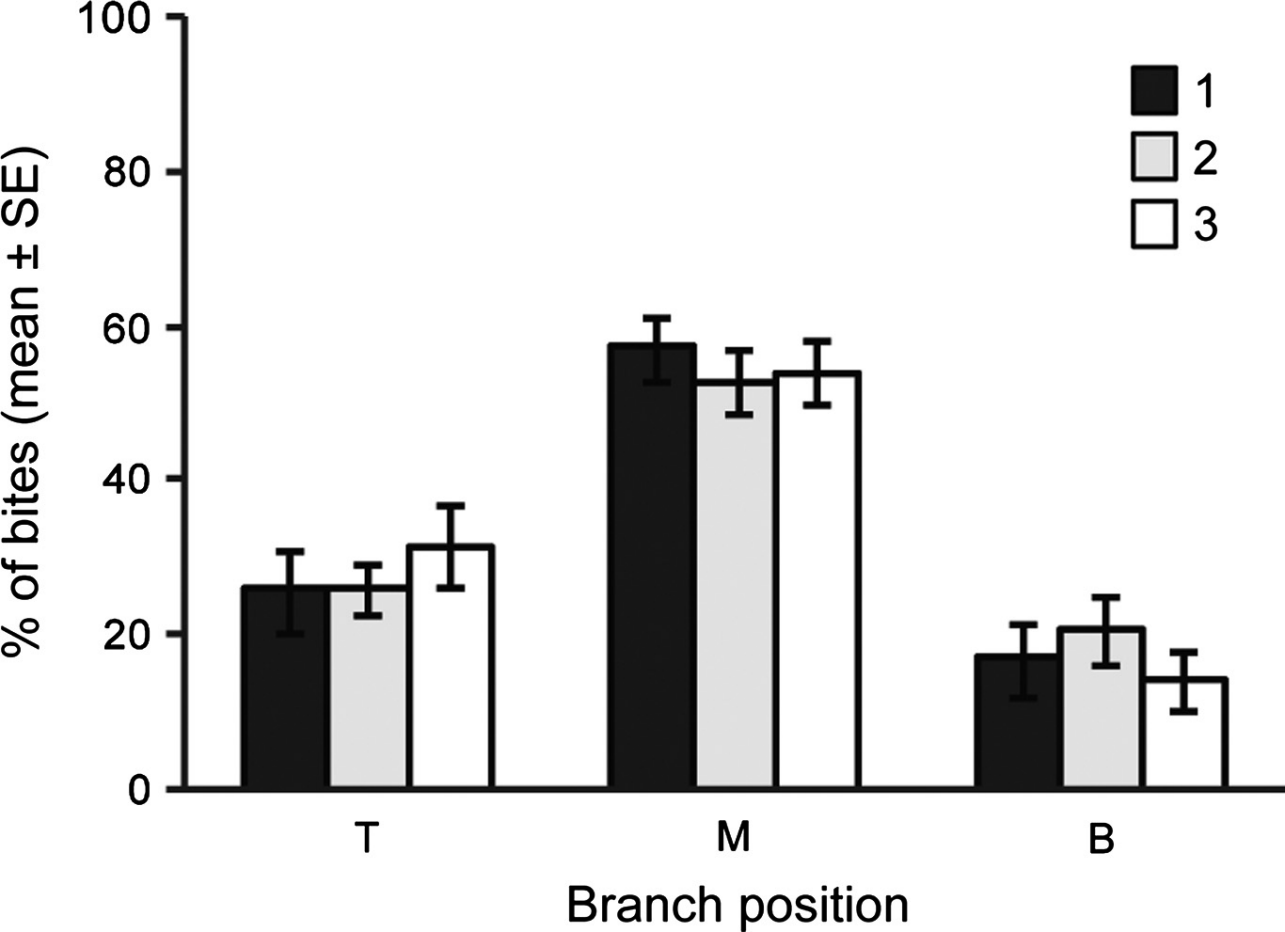
Percentage of total bites (mean ± SE) taken by *Oxymonacanthus longirostris* on each segment of a simulated *Acropora nobilis* branch when artificial corallite extension was consistent along branch. Branch segments are as follows: top (T), middle (M), and base (B). Artificial corallite extensions are as follows: shallow (1), middepth (2), and deep (3). Sample size = 8.

When artificial corallite extension was kept consistent, the percentage of bites was significantly greater in the central segments regardless of the corallite extension length used (all combinations, *P* ≤ 0.001; Fig. 3). No significant difference in percentage of bites was observed between top and base segments under any of the three treatments.

**Figure 4:**
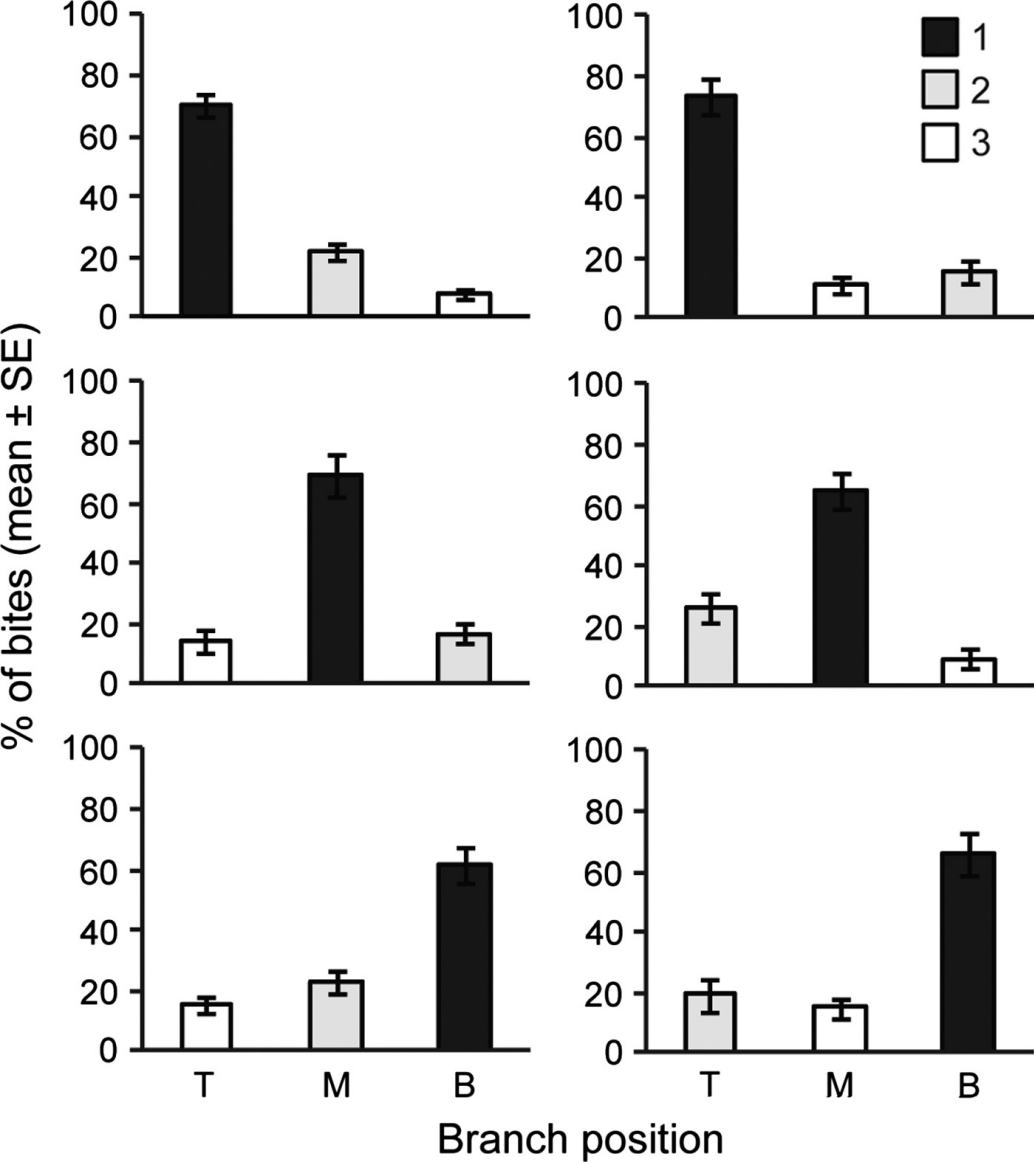
Percentage of total bites (mean ± SE) taken by *Oxymonacanthus longirostris* on each segment of a simulated *Acropora nobilis* branch when artificial corallite extension on each segment varied along branch. Branch segments are as follows: top (T), middle (M), and base (B). Artificial corallite extensions are as follows: shallow (1), middepth (2), and deep (3). Sample size = 8.

When artificial corallite size varied between segments, the percentage of bites also varied significantly between segments (all combinations, *P* < 0.001; Fig. 4). However, variation was related to artificial corallite extension, not to a particular position of a segment. The percentage of bites was significantly higher on the segment with shallow corallites than on either of the other available segments in all six trial combinations. No difference in the percentage of bites was identified between medium or deep segments in any combination.

### Aquarium study of within-colony selectivity

**Figure 5:**
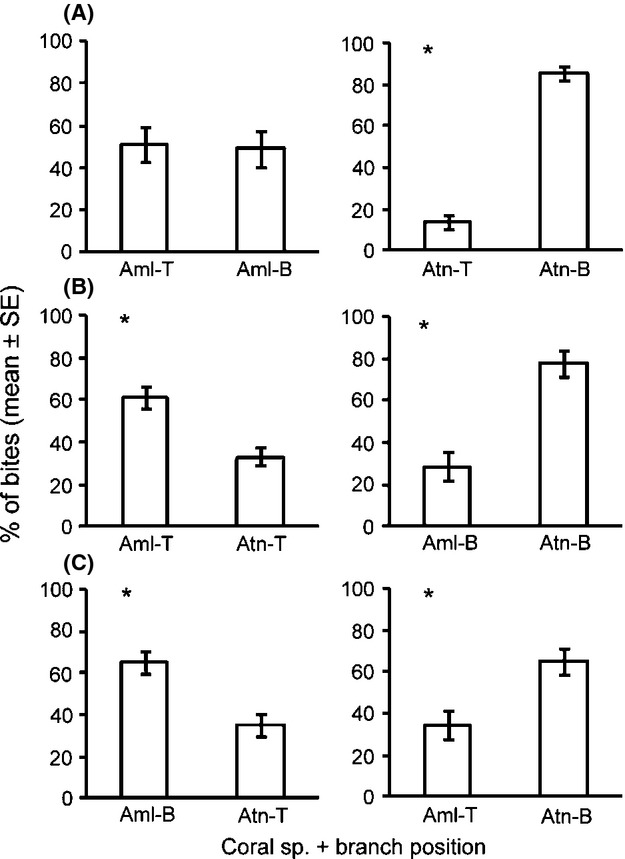
Percentage of total bites taken by *Oxymonacanthus longirostris* on different coral fragments during pairwise trials. Row (A) same coral species but different points of origin (top or base of branch), row (B) different coral species but same points of origin, and row (C) different coral species and different points of origin. Coral fragment types were *Acropora millepora* – base section (Aml-B), *A. millepora* – top section (Aml -T), *Acropora tenuis* – base section (Atn- B), and *A. tenuis* – top section (Atn- T). * indicates a significant difference between means. Number of observations = 16.

No significant preference was identified between *A. millepora* and *A. tenuis* when fish were presented with large pieces of each coral species (*Z* = −0.517, *P* = 0.605). However, during pairwise trials, feeding selectivity varied depending on the choice presented (Fig. 5). While no intraspecific preference was shown between top and bottom sections of *A. millepora* branches (*Z* = −0.155, *P* = 0.877), fish preferentially fed on the bottom sections of *A. tenuis* branches compared with top sections (*Z* = −3.519, *P* < 0.001). When coral species was mixed but the original location (top or bottom sections of branches) was kept the same, preferences between the two coral species depended on whether top or bottom sections were presented. Fish exhibited a preference for top sections of *A. millepora* over top sections of *A. tenuis* (*Z* = −2.482, *P* = 0.013), but preferentially consumed bottom sections of *A. tenuis* over bottom sections of *A. millepora* (*Z* = −2.534, *P* = 0.011). When both species and point of origin of fragments were mixed, fish preferentially fed on the base sections *A. millepora* over top sections of *A. tenuis* (*Z* = −2.327, *P* = 0.02) and base sections of *A. tenuis* over top sections of *A. millepora* (*Z* = −2.068, *P* = 0.039).

### Evaluation of intraspecific morphological variation in corals

**Figure 6:**
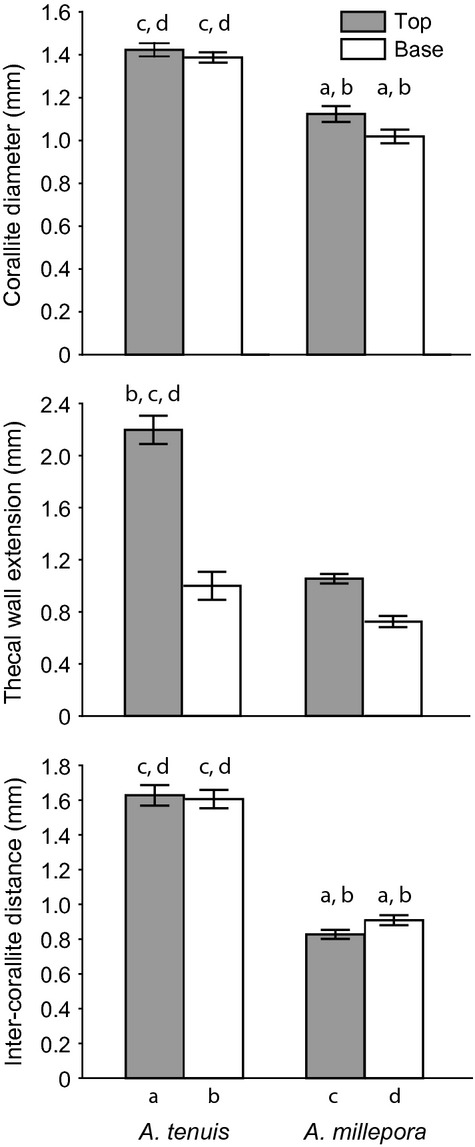
Variation in three selected measures of corallite morphology between coral fragments used in pairwise trials (see Fig. 3). Coral fragment types were *Acropora millepora* – base section, *A. millepora* – top section, *Acropora tenuis* – base section, and *A. tenuis* – top section. Numbers above bars denote the fragments that were significantly different from that fragment for a given measure. Number of coral fragments per type = 12.

No significant differences were identified between sections of *A. nobilis* branches with regard to the morphometric variables recorded (corallite diameter, thecal wall extension, and intercorallite distance), *F*_3,290_ = 1.196, *P* > 0.0005; Wilk's λ = 0.952, partial ε^2^ = 0.02. However, significant morphometric differences were detected within *A. millepora* and *A. tenuis* branches, *F*_3,472_ = 48.6, *P* > 0.0005; Wilk's λ = 0.203, partial ε^2^ = 0.4 (Fig. 6). For corallite diameter, no significant difference was found within *A. millepora* or *A. tenuis*. However, *A. tenuis* corallites were significantly larger than *A*. *millepora* corallites regardless of location (*P* < 0.05). For thecal extension, no significant difference was found within *A. millepora*, or between bottom sections of *A. tenuis* and either *A. millepora* top or bottom sections. However, the thecal wall extension of top sections of *A. tenuis* was significantly higher than all other sections of both species (*P* < 0.05). For intercorallite distance, no difference was found within *A. millepora* or *A. tenuis*. However, intercorallite distance was significantly greater on *A. tenuis* than *A. millepora* regardless of location (*P* < 0.05).

## Discussion

Our field studies and laboratory experiments demonstrate that *O. longirostris* does not feed uniformly from coral colonies, but is selecting feeding positions with greater polyp accessibility, rather than those that are more nutritious. In the field, *O. longirostris* fed nonuniformly on the branching coral, *A. nobilis*, a species that forms the bulk of its diet (Brooker et al. 2013). Fish mostly fed centrally on each coral branch, avoiding areas near the growing tips and bases where branches intercept. Foraging observations also confirmed that *O. longirostris* targets individual polyps. In a pairwise choice experiment, where two factors, *Acropora* species and the point of origin of fragments (top or bottom sections of branches), fish selected fragments with comparatively larger, or numerous polyps. Fish appear to modify their foraging to select the most efficient prey available. When food accessibility was standardized along a branch, fish feed mostly on the central sections of the branch, irrespective of the level of accessibility. However, when food accessibility was manipulated so that it varied along the branch, fish consistently fed on the section of branch with the shallowest corallites regardless of its location. Together, these results suggest that patterns of within-coral selectivity by *O. longirostris* may reflect active choices made to increase foraging efficiency.

The actual tissue consumed by presumed corallivores is often not known (Cole et al. 2008). Our aquarium observations show that *O. longirostris* is predominantly a coral polyp feeder, selectively targeting individual polyps while avoiding the coenosarc. It is likely that this selectivity reflects the relative benefit of coral polyps as a food resource. Each coral polyp consists of a fleshy body cavity extending to the basal plate of the corallite cup, enclosed by the mouth and a ring of tentacles. In contrast, the coenosarc is a relatively thin layer of tissue that covers the underlying skeleton between these polyps. Therefore, selectively targeting polyps should allow a greater volume of tissue to be removed per bite, offsetting any increase in search times. Other corallivores including many butterflyfishes are also assumed to preferentially consume coral polyps (Alwany et al. 2003; Cole et al. 2009). While many species, including *O. longirostris*, have jaw and mouth structures that appear adapted for removing polyps there is limited direct evidence for this, with this assumption often based on gut content analysis that may fail to distinguish between polyps and general tissue (e.g., Hiatt and Strasburg 1960; Sano et al. 1984; Harmelin-Vivien 1989). As *O. longirostris* targets polyps, variation in polyp morphology, defensive structures, or the biochemical composition of polyps that increases or decreases the amount of energy consumed could have a direct influence on prey preferences both within and between coral species. In addition, it is possible that fish target polyps that maximize the effectiveness of their specialized trophic morphology.

In the field, *O. longirostris* exhibited highly nonuniform patterns of foraging on the branching coral *A. nobilis*. Foraging theory predicts individuals should target prey that maximizes energetic return (Pyke et al. 1977). Variation in the tissue composition or surface structure within a coral colony may alter the relative prey value by increasing or reducing the efficiency with which it can be consumed or assimilated. However, no significant variation was found in the morphometric variables of the *A. nobilis* coral branches examined, suggesting that within-colony selectivity is not driven by structural differences of the corallites, at least for this coral species.

There is evidence that the biochemistry of coral tissue can vary within a colony due to metabolic processes. For instance, the concentration of lipid, which is often indicated as being particularly important for corallivorous fishes (Tricas 1989; Rotjan and Lewis 2009), can vary within individual *Acropora* branches. Fang et al. (1989) found that polyps near the growing tip of the branching species, *Acropora formosa,* had lower lipid concentrations than polyps further down the branch, suggesting a biochemical gradient occurs as metabolites are transported up toward the growth point. While this suggests foraging near the growing tip may be less nutritionally beneficial, fish also avoided foraging near the base of branches. Foraging near the base may be less efficient due to the morphological constraints of locating suitable polyps in narrow areas where branches intersect and may require the fish to orientate itself at a suboptimal angle when searching or foraging. Midbranch, fish would have the greatest range of unrestricted motion. It is possible that predation risk may also influence feeding position; feeding near branch tips may increase potential exposure to predators, while feeding near branch bases may restrict movement and escape potential.

Behavioral experiments using simulated *A. nobilis* branches indicated that *O. longirostris* can distinguish between potential prey based on small morphological differences and, when preys are nutritionally similar, will modify their foraging patterns to preferentially select prey that are the most accessible and will presumably require the minimum effort to acquire. When simulated coral branches had identically sized artificial corallites along the branch, making food accessibility equal between segments, fish consistently feed on the central segment. This replicates the foraging patterns observed on *A. nobilis*, a species that morphometric measurements indicated has similar polyp morphology from the base to the tip of the branch. This suggests that, when there is limited structural variation, *O. longirostris* may have an innate drive to feed centrally along the branches of arborecent corals. The underlying basis for this behavior is not known but it may relate to nutritional variation between polyps along a branch if this is consistent between branches (Fang et al. 1989) or morphological constraints that affect foraging efficiency. However, when artificial corallite extension was manipulated this central foraging pattern was overridden, with fish preferentially foraging on the segment with the shallowest artificial corallite size regardless of its location. This consistent modification of foraging selectivity implies that the shallow artificial corallites were the most attractive to fish, either due to food being closer to the surface of the corallite thereby reducing the effort needed to extract it or increasing the amount that could be removed per bite, or food being more visible and so reducing the effort needed to search between bites. This result indicates that foraging decision-making by *O. longirostris* is flexible with fish able to recognize and respond to small differences in prey characteristics and able to modify their foraging behavior when presented with a novel prey to maximize foraging efficiency.

In the pairwise choice experiment using live coral fragments of two preferred *Acropora* species (*A. millepora* and *A. tenuis*), fish varied their prey preferences depending on the combination of coral species and point of origin of fragments (top or bottom sections of branches) presented. The preference patterns observed appear to reflect the morphological differences between coral fragments, specifically those that relate to polyp size and density. For instance, no significant difference was found along *A. millepora* branches with regard to any of the morphological variables recorded, and no foraging preference was exhibited by *O. longirostris*. However, fish exhibited a preference for the lower parts of *A. tenuis* branches where thecal extension was significantly less, and polyps were therefore less protected. Fish also exhibited a general preference for the bottom sections of *A. tenuis* branches over either section of *A. millepora*. While corallite density was slightly higher on *A. millepora*, *A tenuis* was found to have larger corallites. This may increase the relative amount of tissue that can be removed per bite, increasing overall foraging efficiency (Tricas 1989). No preference was observed between coral species when fish were provided with larger sections of coral composed of several whole branches. As *A. millepora* is known to be a preferred prey for *O. longirostris* (Brooker et al. 2013), it is therefore possible that overall both species represent equally valuable prey for these fishes. However, *A. millepora* may still be preferentially selected in the wild as fish chose the upper sections of *A. millepora* over those of *A. tenuis*, and lower sections of *A. tenuis* branches would remain difficult to access within fully intact colonies. The relationship between variation in corallite structure and prey preferences suggests that small-scale morphological differences between and within corals can affect the foraging decisions of *O. longirostris*.

Foraging selectivity is exhibited in many corallivorous species (Cole et al. 2008) with the consumption of preferred coral having beneficial effects on a variety of fitness-related parameters (Berumen et al. 2005; Berumen and Pratchett 2008; Brooker et al. 2013). It is generally assumed that these preferences relate to the nutritional content of coral tissue (Berumen et al. 2011; Pisapia et al. 2012). Despite this, attempts to relate preferences for specific corals to the relative levels of the major energetic macronutrients, such as lipids, protein, and carbohydrates, have failed to find strong correlations (Tricas 1989; Keesing 1990). However, these studies have generally considered the biochemical profile of each sampled colony as a single replicate. When within-colony differences were assessed, namely the total reproductive effort of polyps, Rotjan and Lewis (2009) found parrotfish consumed areas of *Montastraea* colonies with high numbers of gametes, ostensibly due to their higher protein and lipid levels. If the nutritional value of coral tissue consistently varies within a colony, and corallivores only target specific parts, then relevant differences in nutritional quality between coral species may have failed to be recognized due to a sampling methodology that does not account for these within-colony foraging patterns. Future work should therefore consider the biochemical variation within corals when attempting to determine a nutritional basis for foraging preferences.

Our results provide strong support for the hypothesis that coral morphology can influence corallivore foraging preferences. Morphology has previously been indicated in the preferences of the butterflyfish, *C. multicinctus*, where fish exhibited a strong preference for the massive *Porites lobata* over the branching *Porites compressa* (Tricas 1989), implying that the relatively flat foraging surface of *P. lobata* was the key driver of the preference. Many corallivorous fishes, including *O. longirostris*, preferentially target morphologically similar *Acropora* corals, generally digitate species with short branches and a relatively open corallite structure (Cole et al. 2008; Brooker et al. 2013). These corals may allow fish to ingest a relatively large amount of tissue per bite while requiring limited reorientation between bites. It is therefore possible that for ecologically similar corallivores, such as many butterflyfishes, coral morphology may also play a key role in determining dietary preferences. While it is likely that a variety of interacting factors influence the foraging preferences of these species, further work that determines the relative importance of nutritional quality versus accessibility may help to decipher why corallivores prefer certain corals.

In conclusion, our study shows that this corallivorous fish is a highly selective polyp feeder, with within-colony feeding selectivity probably driven by a combination of both innate preferences and responses to small-sale differences in polyp morphology that may affect foraging efficiency. *Acropora* corals appear to be highly variable in their value as prey and this can affect condition and fitness of individuals (Berumen and Pratchett 2008; Brooker et al. 2013). As obligate corallivores must achieve a nutritional balance from within a relatively narrow range of potential prey, precise behavioral mechanisms that increase foraging efficiency may help these species to maximize their performance.
